# Protective Effects of Gintonin on Reactive Oxygen Species-Induced HT22 Cell Damages: Involvement of LPA1 Receptor-BDNF-AKT Signaling Pathway

**DOI:** 10.3390/molecules26144138

**Published:** 2021-07-07

**Authors:** Yeon-Jin Cho, Sun-Hye Choi, Ra-Mi Lee, Han-Sung Cho, Hyewhon Rhim, Hyoung-Chun Kim, Byung-Joo Kim, Jong-Hoon Kim, Seung-Yeol Nah

**Affiliations:** 1 Ginsentology Research Laboratory and Department of Physiology, College of Veterinary Medicine, Konkuk University, Seoul 05029, Korea; yeonjin0202@naver.com (Y.-J.C.); vettman@naver.com (S.-H.C.); rmlee12@konkuk.ac.kr (R.-M.L.); newlove0202@nate.com (H.-S.C.); 2 Center for Neuroscience, Korea Institute of Science and Technology, Seoul 02792, Korea; e-hrhim@kist.re.kr; 3 Neuropsychopharmacology and Toxicology Program, College of Pharmacy, Kangwon National University, Chunchon 24341, Korea; kimhc@kangwon.ac.kr; 4 Division of Longevity and Biofunctional Medicine, Pusan National University School of Korean Medicine, Yangsan 50612, Korea; vision@pusan.ac.kr; 5 Biosafety Research Institute, College of Veterinary Medicine, Chonbuk National University, Baekje-daero 567, Jeonju 28644, Korea; jhkim1@jbnu.ac.kr

**Keywords:** ginseng, gintonin, hippocampal cell line, oxidative stress, BDNF/TrkB/Akt, neuroprotection

## Abstract

Gintonin is a kind of ginseng-derived glycolipoprotein that acts as an exogenous LPA receptor ligand. Gintonin has in vitro and in vivo neuroprotective effects; however, little is known about the cellular mechanisms underlying the neuroprotection. In the present study, we aimed to clarify how gintonin attenuates iodoacetic acid (IAA)-induced oxidative stress. The mouse hippocampal cell line HT22 was used. Gintonin treatment significantly attenuated IAA-induced reactive oxygen species (ROS) overproduction, ATP depletion, and cell death. However, treatment with Ki16425, an LPA1/3 receptor antagonist, suppressed the neuroprotective effects of gintonin. Gintonin elicited [Ca^2^⁺]_i_ transients in HT22 cells. Gintonin-mediated [Ca^2^⁺]_i_ transients through the LPA1 receptor-PLC-IP_3_ signaling pathway were coupled to increase both the expression and release of BDNF. The released BDNF activated the TrkB receptor. Induction of TrkB phosphorylation was further linked to Akt activation. Phosphorylated Akt reduced IAA-induced oxidative stress and increased cell survival. Our results indicate that gintonin attenuated IAA-induced oxidative stress in neuronal cells by activating the LPA1 receptor-BDNF-TrkB-Akt signaling pathway. One of the gintonin-mediated neuroprotective effects may be achieved via anti-oxidative stress in nervous systems.

## 1. Introduction

Ginseng, the root of Panax ginseng Meyer, is a well-known folk medicine used in traditional herbal medicine in Korea. Panax ginseng has been used as a tonic for many centuries [1]. Ginseng contains ginsenosides, ginseng polysaccharides, fatty acids, lipids, and other minor components [2]. Ginsenosides (called ginseng saponins) are one of the representative bioactive components of ginseng and have anti-inflammatory, anticancer, anti-fatigue, antioxidant, and neuroprotective effects [3]. Ginseng polysaccharides exhibit immunostimulatory activity through macrophage stimulation [4]. However, the identified components of ginseng do not fully explain the molecular mechanisms of diverse ginseng pharmacology [2].

Recent studies have demonstrated that ginseng, including ginseng root, leaf, and stem, contains gintonin, which is a non-saponin and non-polysaccharide [5]. The main functional ingredients of gintonin are lysophosphatidic acids (LPAs), such as LPA C18:2, LPA C16:0, and LPA C18:1, in which LPA C18:2 is more abundant [6]. Gintonin is a ligand for the GTP-binding protein-coupled lysophosphatidic acid (LPA) receptor and activates the LPA receptors, which induces [Ca^2+^]_i_ transients from the endoplasmic reticulum [7]. Gintonin mainly activates Gα_q/11_ and Gα_12/13_ and initiates downstream signaling through phospholipase C (PLC) and Rho kinase activation [8]. Gintonin-mediated [Ca^2+^]_i_ transients stimulate the release of neurotransmitters, such as acetylcholine, dopamine, and glutamate, to facilitate synaptic transmission via the LPA receptor regulations [9]. The gintonin-LPA receptor signaling pathway further improves cognitive functions and is effective in aging-related neurodegenerative diseases, such as Alzheimer’s disease (AD), Parkinson’s disease (PD), and Huntington’s disease [10].

Oxidative stress is caused by increased reactive oxygen species (ROS) production. Endogenous antioxidant activity in the hippocampus is reduced because of aging, resulting in ischemia and neurodegenerative diseases [11]. In contrast, iodoacetic acid (IAA) is an irreversible inhibitor of the glycolytic enzyme glyceraldehyde 3-phosphate dehydrogenase (GAPDH). IAA can lower cellular glycolytic activity, which leads to decreased ATP production, increased ROS levels, loss of membrane potential, and mitochondrial dysfunction, finally leading to neuronal cell death [12]. These changes following treatment with IAA in neuronal cells are similar to those observed in experimental animal models of ischemic stroke [13]. Therefore, IAA is used as a model compound to mimic in vitro hypoxic/ischemia conditions in neurons [14].

In previous in vivo studies, we demonstrated that oral administration of gintonin or gintonin-enriched fraction increased the expression of proteins involved in learning and memory and that of genes that affect cognitive function via anti-neurodegenerative activities [15]. However, relatively little is known about the neuroprotective effects of gintonin against oxidative stress in neuronal cells. In the present study, we studied the neuroprotective effect of gintonin on IAA-induced HT22 cell, a hippocampal cell line, oxidative stress and its molecular mechanism of action. Finally, gintonin showed neuroprotective effects via the LPA1 receptor/PLC/IP_3_-dependent BDNF/TrkB/Akt signaling pathway in HT22 cells.

## 2. Results

### 2.1. Gintonin Attenuates IAA-Induced Cell Damages in HT22 Cells

**Figure 1:**
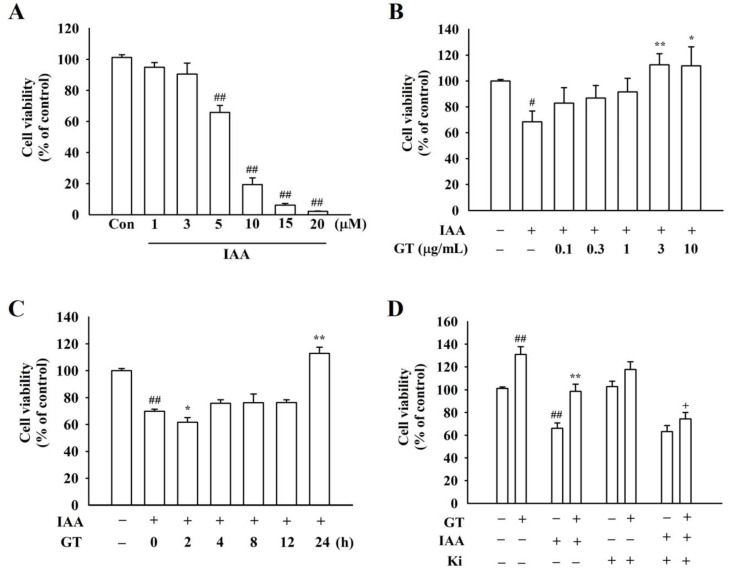
Effects of gintonin on cell viability against iodoacetic acid (IAA)-mediated HT22 cell death. (**A**) IAA-induced cell death in a dose-dependent manner in HT22 cells treated with multiple concentrations of IAA (1, 3, 5, 10, 15, and 20 μM) for 2 h. (**B**) Dose-dependent effect of gintonin on cell viability against IAA-mediated cell apoptosis. After treating with 5 μM IAA for 2 h, HT22 cells were exposed to various concentrations of gintonin (0.1, 0.3, 1, 3, and 10 μg/mL) for 24 h. (**C**) Time-dependent effect of gintonin on cell viability against IAA-mediated cell apoptosis. After treating with 5 μM IAA for 2 h, HT22 cells were exposed to gintonin (3 μg/mL) at various times (2, 4, 8, 12, and 24 h). (**D**) Involvement of LPA1/3 receptors in gintonin-mediated cell protection against IAA. After treating with 5 μM IAA for 2 h, HT22 cells were pretreated with Ki16425 (final concentration 10 μM), an LPA1/3 receptor antagonist, for 30 min and then co-treated with 3 μg/mL gintonin for 24 h. Cell viability was estimated with the XTT assay kit. The data are represented as the mean ± standard error of the mean (SEM; *n* = 5). Statistical significances were determined by either one-way ANOVA (**A**–**C**) or three-way ANOVA (**D**). ^#^ *p* < 0.05 and ^##^ *p* < 0.01 compared to the control group; * *p* < 0.05 and ** *p* < 0.01 compared to the IAA-treated group; ^+^ *p* < 0.01 compared to the GT + IAA group.

To determine the in vitro cytotoxicity of IAA, HT22 cells were treated with IAA at various concentrations. IAA induced cell damages in a dose-dependent manner (*p* < 0.01, compared to the control group, Figure 1A). IAA was used at 5 μM for 2 h, as this concentration is suitable for the following experiments. Gintonin prevented IAA-induced cell damages in a dose-dependent manner and showed a peak effect in HT22 cells at 3 μg/mL (Figure 1B). In a time-dependent study, gintonin-mediated neuroprotective effects of gintonin against IAA were observed after 24 h (Figure 1C). However, pretreatment with 10 μM Ki16425, an LPA1/3 receptor antagonist, blocked gintonin-mediated attenuation of IAA-induced cell death (Figure 1D). These results indicate that gintonin significantly attenuates IAA-induced cytotoxicity through LPA1/3 receptors.

### 2.2. Gintonin Suppresses IAA-Induced ROS Production

**Figure 2:**
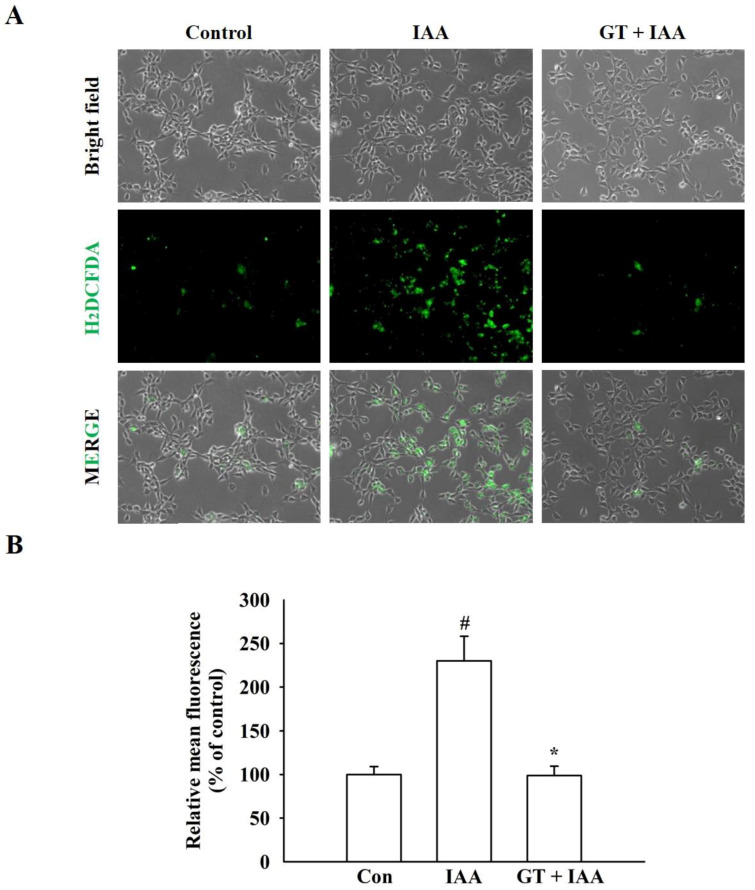
Effect of gintonin on IAA-mediated ROS accumulation in HT22 cells. (**A**) Representative images of CM-H2DCFDA staining. HT22 cells were pretreated with or without gintonin (3 μg/mL) for 1 h, washed, and treated with 5 μM IAA for another 2 h. The cells were exposed to CM-H2DCFDA (10 μM) for 30 min. Microscopy images were captured at the same magnification, scale bar = 100 μm. (**B**) Quantitative analysis of the images obtained from fluorescence microscopy. The data are represented as the mean ± standard error of the mean (SEM; *n* = 4). ^#^ *p* < 0.01 compared to the control group; * *p* < 0.01 compared to the IAA alone-treated group.

Since IAA induces cell death due to the overproduction of ROS [14], CM-H2DCFDA, an oxidant-sensitive dye, was used to determine whether gintonin could decrease IAA-induced intracellular ROS formation. As shown in Figure 2A,B, IAA treatment significantly increased intracellular ROS production compared to that in the control group, but gintonin treatment significantly reduced IAA-induced ROS production to near the control level. These results show that gintonin-mediated neuronal cell protection after IAA treatment is due to a reduction in IAA-induced ROS production (Figure 2).

### 2.3. Gintonin Prevents against IAA-Induced ATP Depletion

To determine whether the neuroprotective effect of gintonin against IAA-induced cell death is due to the restoration of cellular ATP content, intracellular ATP levels were measured in the presence of IAA. IAA treatment (5 μM, 2 h) of HT22 cells significantly reduced intracellular ATP concentration by 40% compared to that in control cells. Gintonin treatment prevented ATP reduction in IAA-treated HT22 cells. Gintonin-mediated ATP restoration from IAA damage was also inhibited by Ki16425, an LPA1/3 receptor antagonist. Moreover, gintonin alone increased the ATP content. Thus, these results indicate that gintonin restores intracellular ATP levels via the LPA1 receptor (Figure S1, Supplementary Materials).

### 2.4. Gintonin Increases BDNF Expression under IAA Insult

**Figure 3:**
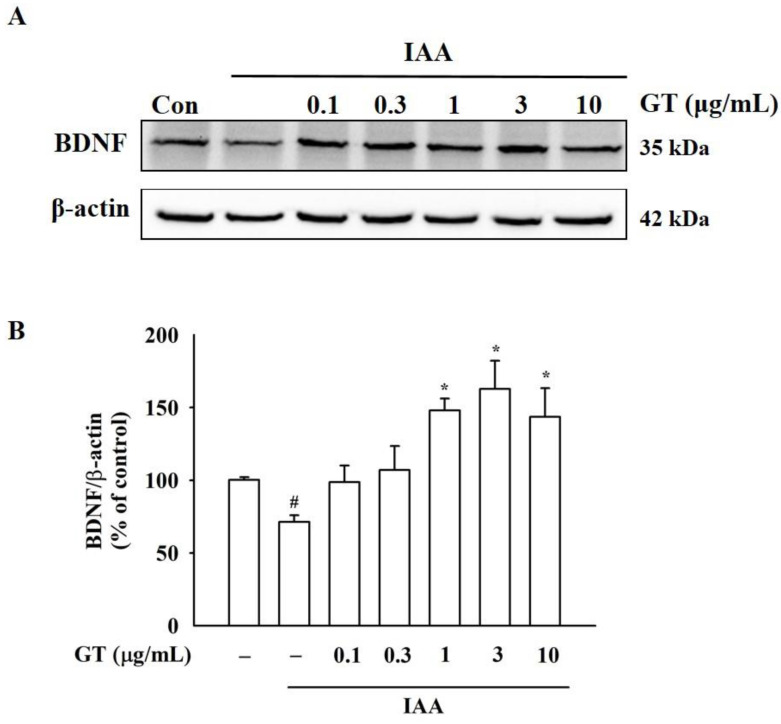
Changes in brain-derived neurotrophic factor (BDNF) protein expression induced by gintonin in IAA-treated HT22 cells. (**A**) BDNF expression levels were detected by Western blot analysis using β-actin as a loading control. HT22 cells were treated with 5 μM IAA for 2 h, followed by gintonin treatment with different concentrations (0.1–10 μg/mL) for 24 h in the absence or presence of IAA. (**B**) BDNF/β-actin ratio at each dose. The data are represented as the mean ± standard error of the mean (SEM; *n* = 4). ^#^ *p* < 0.01 compared to the control group; * *p* < 0.01 compared to the IAA-treated group.

BDNF, a typical brain neurotrophic factor, plays a critical role in maintaining brain homeostasis, including neuronal survival under insults of ROS [16]. Next, the effect of IAA on BDNF expression and level was examined. As shown in Figure S2A, Supplementary Materials, IAA treatment (5 μM, 2 h) reduced cytoplasmic BDNF expression compared to the control group, as determined by immunocytochemical analysis. However, co-treatment of gintonin with IAA restored cytosolic BDNF expression, and treatment with gintonin alone also increased BDNF expression (Figure S2B, Supplementary Materials). In Western blot analysis, although IAA alone treatment decreased BDNF protein expression, co-treatment of cells with 3 μg/mL gintonin in the presence of IAA increased BDNF levels (Figure 3A,B). According to immunocytochemical staining and Western blot results, gintonin restored the IAA-induced attenuation of BDNF expression (Figure 3 and Figure S2, Supplementary Materials), indicating the involvement of BDNF in gintonin-mediated attenuation of IAA-induced neuronal cell damage.

### 2.5. Gintonin Induces [Ca^2+^]_i_ Transients and Its Signal Transduction Pathway

In a previous study, we have shown that gintonin induces cellular responses, including cell proliferation and neurotransmitter release through [Ca^2+^]_i_ transients [2]. Gintonin treatment also induced [Ca^2+^]_i_ transients in a concentration-dependent manner in HT22 cells (Figure S3A, Supplementary Materials). The presence of IAA attenuated gintonin-mediated [Ca^2+^]_i_ transients compared to treatment with gintonin alone, although gintonin still increased [Ca^2+^]_i_ transients in a dose-dependent manner (Figure S3B,C, Supplementary Materials). Gintonin-mediated [Ca^2+^]_i_ transients were significantly attenuated by the LPA1/3 receptor antagonist, Ki16425, and the phospholipase C inhibitor, U73122. Gintonin-mediated [Ca^2+^]_i_ transients were completely blocked by the inositol 1,4,5-triphosphate (IP3) receptor antagonist, 2-APB, and the intracellular Ca^2+^ chelator, BAPTA-AM (Figure S3D,E, Supplementary Materials). Thus, these results indicate that gintonin induces [Ca^2+^]_i_ transients through activation of the LPA1/3 receptor-phospholipase C-IP_3_ receptor-[Ca^2+^]_i_ transient signaling transduction pathway.

### 2.6. Gintonin Ameliorates IAA-Induced Inhibition of BDNF Release

**Figure 4:**
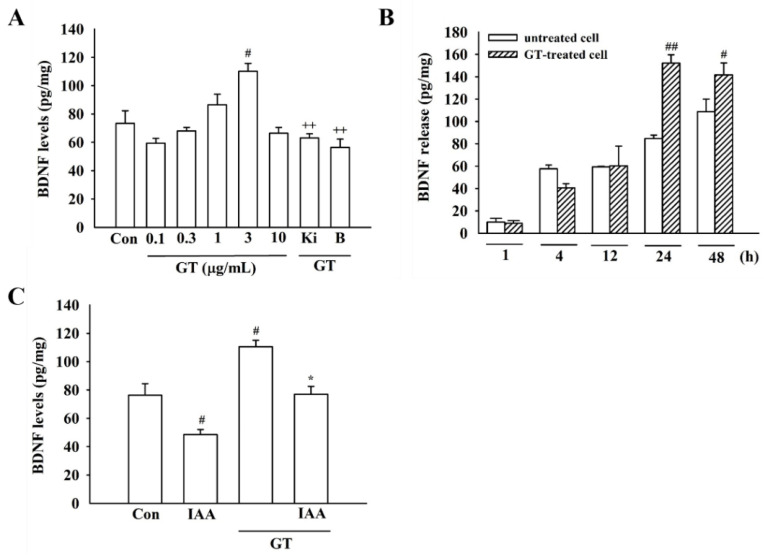
Effect of gintonin on the amount of BDNF release under IAA insult in HT22 cells. (**A**) The levels of BDNF released from HT22 cells treated with the indicated concentrations (0.1–10 μg/mL) of gintonin for 24 h or with gintonin (3 μg/mL) in the presence of Ki16425 (Ki) (10 μM, 30 min) and BAPTA-AM (50 μM, 30 min). (**B**) HT22 cells were exposed to 3 μg/mL gintonin at various times (1, 4, 12, 24, and 48 h). (**C**) Effects of gintonin (3 μg/mL, 24 h) on BDNF release in the presence of IAA (5 μM, 2 h). The data are represented as the mean ± standard error of the mean (SEM; *n* = 4). ^#^ *p* < 0.05 and ^##^ *p* < 0.01 compared to control group; * *p* < 0.05 compared to IAA-treated group; ^++^ *p* < 0.01 compared to the gintonin-treated group.

Since co-treatment of gintonin with IAA restored BDNF expression in immunocytochemical and Western blotting analyses, gintonin effects on BDNF release in the absence or presence of IAA were quantified. Gintonin alone (3 μg/mL, 24 h) increased BDNF release in a concentration- and time-dependent manner. Gintonin-induced BDNF release was blocked by Ki16425, an LPA1/3 receptor antagonist, and BAPTA-AM, an intracellular calcium chelator (Figure 4A,B). This indicates the involvement of the LPA1 receptor and [Ca^2+^]_i_ transients in gintonin-mediated BDNF release. Next, IAA treatment inhibited BDNF release in cells, but co-treatment with gintonin and IAA restored BDNF release to the control level (Figure 4C), showing that gintonin might help to overcome IAA-induced inhibition of BDNF expression and release.

### 2.7. Gintonin Stimulates the TrkB/Akt Signaling Pathway through BDNF Release

**Figure 5:**
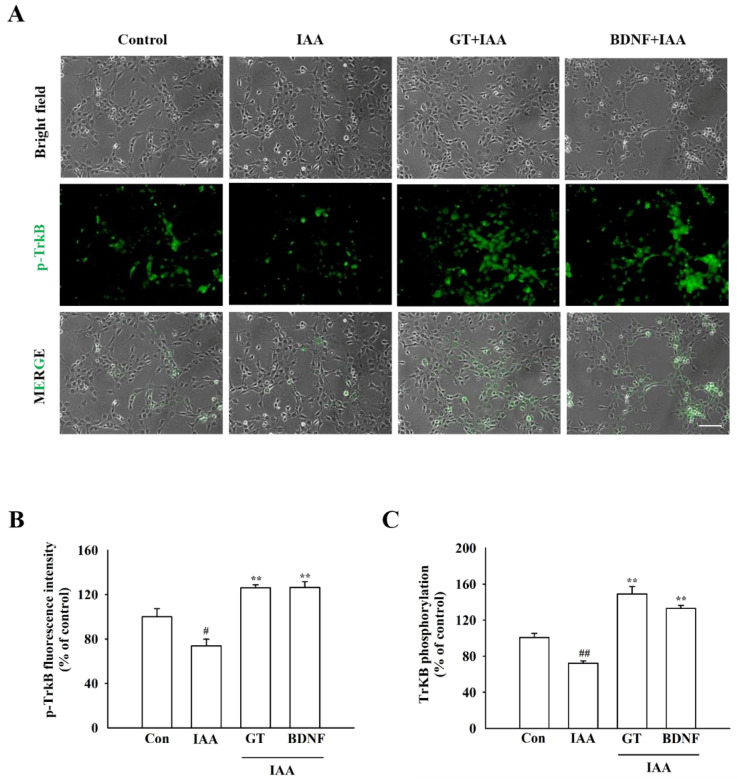
Effect of gintonin on the activation of TrkB receptors in IAA-treated HT22 cells. (**A**) Representative microscopy images of TrkB phosphorylation. HT22 cells were exposed to IAA (5 μM, 2 h) and treated with gintonin (3 μg/mL, 24 h) or BDNF (30 ng/mL, 24 h). Phosphorylated TrkB is dyed green. Microscopy images were captured at the same magnification, scale bar = 100 μm. (**B**) Quantitative analysis of the images obtained from fluorescence microscopy. (**C**) The level of phosphorylation of TrkB receptors measured using a phosphor-TrkB ELISA kit. The data are expressed as the mean ± standard error of the mean (SEM; *n* = 4). ^#^ *p* < 0.05 and ^##^ *p* < 0.01 compared to the control group; ** *p* < 0.01 compared to the IAA-treated group.

**Figure 6:**
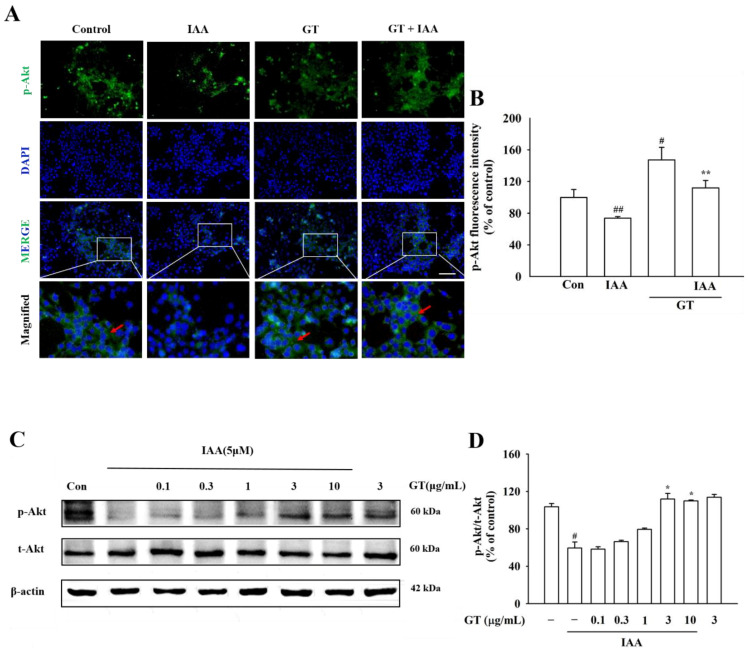
Effect of gintonin on Akt phosphorylation in the presence of IAA in HT22 cells. (**A**) Representative microscopy images of Akt phosphorylation. The phosphorylation of Akt was detected using immunochemistry staining. HT22 cells were treated with IAA (5 μM, 2 h), followed by exposure to gintonin (3 μg/mL, 24 h). Phosphorylated TrkB is dyed green, and nuclei are dyed blue using DAPI. Microscopic images were captured at the same magnification, scale bar = 100 μm. (**B**) Quantitative analysis of the images obtained from fluorescence microscopy. (**C**) The expression of p-Akt and t-Akt proteins was detected by Western blot analysis using β-actin as a loading control. (**D**) p-Akt/t-Akt ratio at each dose. The data are represented as the mean ± SEM (*n* = 4). ^#^ *p* < 0.05 and ^##^ *p* < 0.01 compared to control group; * *p* < 0.05 and ** *p* < 0.01 compared to the IAA-treated group.

**Figure 7:**
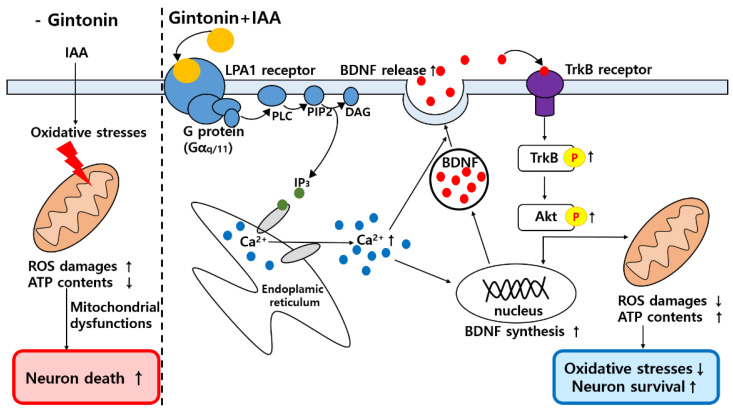
Schematic diagram of gintonin-mediated neuroprotective activity via LPA1 receptor activation under insults of oxidative stressor, IAA, in HT22 cells. Gintonin reduces reactive oxygen species (ROS) levels produced by IAA and has neuroprotective effects via LPA1 receptor-BDNF-TrkB-Akt signaling pathway.

To determine whether the gintonin-induced release of BDNF reduced IAA-induced oxidative stress through the TrkB/Akt signaling pathway, HT22 cells were treated with gintonin after IAA exposure. Immunocytochemistry and phospho-TrkB ELISA kit showed that TrkB phosphorylation decreased in IAA-treated HT22 cells, compared to that in the control group (Figure 5A,B). However, gintonin treatment ameliorated the decrease in TrkB phosphorylation in IAA-treated HT22 cells. Gintonin increased the phosphorylation of TrkB to a level similar to that of BDNF treatment (30 ng/mL) used as a positive control (Figure 5C). Immunochemistry and Western blot analyses results showed that IAA treatment also decreased Akt phosphorylation compared to that in the control group. Treatment with gintonin restored Akt phosphorylation in Western blotting, which was reduced by IAA treatment (Figure 6A–D). Taken together, these results indicate that BDNF released by gintonin activates the TrkB/Akt signaling pathway to reduce the oxidative stress induced by IAA (Figure 7).

## 3. Discussion

HT22 cells are sub-cloned cell line from HT-4 cells, which are immortalized primary hippocampal neurons [17,18,19]. Various studies including antidepressant and other natural products was performed using HT22 cells [18,20,21]. The LPA1 receptor is highly expressed in HT22 hippocampal cells and is a key receptor for serum-deprivation induced cell death [18]. Thus, HT22 cell is suitable as a neuronal model cell for identifying molecular mechanisms associated with the neuroprotective effect of gintonin. However, relatively little is known about the neuroprotective effects of gintonin on in vitro oxidative stress in HT22 cells. In the present study, we demonstrated that gintonin attenuated IAA-induced in vitro oxidative stress via the LPA1/3 receptor/PLC/IP_3_-dependent BDNF/TrkB/Akt signaling pathway in HT22 cells.

Mitochondria are the main organelles that play a key role in cellular energy metabolism, including ATP production [22]. ATP is supplied through glycolysis or oxidative phosphorylation to maintain cellular homeostasis by inhibition of ROS accumulation [23]. IAA induces chemical hypoxia by blocking GAPDH, inhibits glycolysis, and damages mitochondria by inhibiting mitochondrial ATP production [24]. Mitochondria impaired by IAA produce less ATP, but more ROS [22]. Thus, oxidative stress induced by ROS overproduction is an important risk factor for pathological progression of neurodegenerative diseases, such as AD and PD [25]. Changes, such as mitochondrial damage, ATP loss, and increased ROS production, trigger cell death and neurodegenerative diseases [22]. A compound that has neuroprotective effects against the aforementioned deteriorating conditions by IAA could be a target for new drug development for these diseases. In the present study, the neuroprotective effects of gintonin on oxidative stress were investigated using IAA-treated HT22 cells. Gintonin treatment could provide neuronal cell protection against IAA-induced hippocampal cell death, ROS increase, and ATP reduction through the LPA1 receptor (Figure 1 and Figure 2 and Figure S1, Supplementary Materials). In addition, gintonin itself also showed proliferative effects of HT22 cells even in the absence of IAA. This effect was also observed in previous reports in neuronal and non-neuronal cells [26,27] and might be due to innate properties of LPA receptors [28].

The molecular mechanisms of gintonin-mediated neuroprotective effects against IAA-induced oxidative stress remain unclear. In the present study, gintonin might exhibit neuroprotective effects through a three-step process. First, gintonin stimulates BDNF release and increases BDNF level through [Ca^2+^]_i_ transients after activation of the LPA1/3 receptor signaling pathway (Figure 3 and Figure 4 and Figures S2 and S3, Supplementary Materials). Previous studies have demonstrated that gintonin-induced [Ca^2+^]_i_ transients regulate the release of neurotransmitters, such as acetylcholine, dopamine, glutamate, and VEGF, in neurons and astrocytes, through the LPA1 receptor [2]. The released acetylcholine and glutamate affect the hippocampal cholinergic system and hippocampal synaptic transmission, respectively, showing the molecular basis of the pharmacological effects of gintonin on cognitive and neuroprotective functions [2]. Similarly, gintonin treatment stimulated BDNF release and synthesis (Figure 3 and Figure 4). BDNF is a neurotrophic factor that is essential for brain homeostasis (i.e., neurogenesis, neuronal survival, and cognitive impairment from various insults such as ROS) in the hippocampus [29]. BDNF is highly expressed in the hippocampus, which is also related to learning, memory, and cognitive functions [30]. Furthermore, BDNF protects neuronal cells from oxidative stress [31]. To increase neuronal survival, GTP-binding protein-coupled receptor (GPCR)-mediated [Ca^2+^]_i_ transients phosphorylate CREB through phosphorylation of several kinases, including calmodulin-dependent protein kinase (CaMK), and stimulate BDNF transcription [30]. Thus, gintonin-mediated BDNF release and synthesis via LPA receptors in HT22 cell may contribute to the anti-ROS activity.

Second, BDNF released by gintonin-induced [Ca^2+^]_i_ transients might bind and activate TrkB to phosphorylate TrkB (Figure 5 and Figure 6). BDNF binding to TrkB is coupled with the attenuation of oxidative stress-induced cell death [32]. Gintonin-induced BDNF/TrkB signaling pathway is linked to Akt phosphorylation (Figure 6 and Figure 7). Thus, the last proposal is that activated Akt also might stimulate the expressions of Nrf2/HO1 protein to attenuate IAA-induced oxidative stress, supporting this notion that in previous reports we showed that gintonin increased the Nrf2/HO1 expressions under oxidative stress [33,34,35]. Additionally, phosphorylated Akt regulates other essential cellular responses, such as cell growth, proliferation, survival under mitochondrial dysfunctions, and attenuation of cell death [36]. Thus, the BDNF/TrkB/Akt axis protects against ROS-induced cell death [36,37]. However, PKC pathway is not involved in gintonin rescue against IAA (data not shown). The BDNF released by gintonin activates the Akt signaling pathway to enhance mitochondrial function and neuronal cell survival in HT22 cells (Figure 7).

Although IAA used in the present study can be assumable as an experimental model, it has several limitations as a ROS model system. Thus, IAA also inhibits several enzymes in many cells and biological pathways, since it is an inhibitor of cysteine-dependent enzymes at the active site [38]. In addition to glycolysis and specific GAPDH inhibition, it also induces in vivo toxicity [39]. In future, it might require further proof on gintonin effects against ROS using another model system.

In summary, we showed that gintonin treatment attenuated IAA-induced HT22 cell death. Gintonin treatment increases the expression and amount of BDNF release by inducing [Ca^2+^]_i_ transients from the endoplasmic reticulum through the LPA1/3 receptor-PLC-IP_3_ signaling pathway. The released BDNF binds to TrkB to phosphorylate TrkB and then phosphorylates Akt. Phosphorylated Akt affects BDNF synthesis and improves mitochondrial function. Thus, gintonin exhibits neuroprotective effects against IAA-induced oxidative stress, which may serve as a molecular basis for treatment against in vivo neurodegenerative diseases.

## 4. Materials and Methods

### 4.1. Gintonin Preparation from Ginseng

Gintonin (GT) was prepared from Panax ginseng according to a previously described method [5].

### 4.2. Cell Culture

HT22 cells were purchased from Sigma-Aldrich (St. Louis, MO, USA). HT22 cells were cultured in Dulbecco’s modified Eagle’s medium (DMEM) supplemented with 10% heat-inactivated fetal bovine serum (FBS), 100 μg/mL streptomycin, and 100 units/mL penicillin at 37 °C with 5% CO_2_.

### 4.3. Measurement of Cell Viability

Cell viability was measured using a WST-8-(2-(2-methoxy-4-nitrophenyl)-3-(4-nitrophenyl)-5-(2,4-disulfophenyl)-2H-tetrazolium, monosodium salt) (XTT) assay according to the manufacturer’s protocol. Briefly, HT22 cells were seeded at 5 × 10^3^ cells/well/100 μL into 96-well plates and incubated overnight at 37 °C. The next day, the cells were washed with serum-free DMEM and treated with 5 μM iodoacetic acid (IAA). After 2 h of incubation, the medium containing IAA was removed and replaced with fresh medium in the presence or absence of gintonin for 24 h. Cells were pretreated with Ki16425, an LPA 1/3 receptor antagonist, 30 min before gintonin treatment and co-treated with gintonin for 24 h. The medium was changed with 90 μL of serum-free DMEM without phenol red and 10 μL of XTT reaction solution. After 2 h of incubation, the absorbance was measured at 450 nm using a microplate reader (Molecular Devices, San Jose, CA, USA).

### 4.4. Measurement of Intracellular ROS Levels

To measure intracellular ROS in HT22 cells, the non-fluorescent compound CM-H2DCFDA (Invitrogen, Carlsbad, CA, USA) was used. CM-H2DCFDA is an ROS indicator that permeates the cell membrane and exhibits fluorescence when the acetate groups are removed by intracellular esterase or upon cellular oxidation. HT22 cells were seeded at 2 × 10^5^ cells/mL on 10 mm glass coverslips coated with poly-l-lysine (PLL). The next day, the cells were pretreated with 3 μg/mL gintonin for 1 h. Then, the cells were washed with serum-free DMEM and treated with 5 μM IAA. After 2 h, the medium was aspirated and replaced with 10 μM CM-H2DCFDA in serum-free DMEM without phenol red for 30 min at 37 °C in the dark. Then, the cells were washed with PBS three times and fixed in 4% paraformaldehyde in PBS for 20 min at room temperature in the dark. After three washes with PBS, the cells were stained for nuclei and mounted with Vectashield Mounting Media (Vector Laboratories, Burlingame, CA, USA) containing 4′,6-diamidino-2-phenylindole (DAPI). The fluorescent images were captured with an Axio200 inverted fluorescence microscope (Carl Zeiss, Oberkochen, Baden-Württemberg, Germany) using a green fluorescence filter.

### 4.5. Measurement of Brain-Derived Neurotrophic Factor (BDNF) Release Concentration from HT22 Cells

To measure the concentration of BDNF released from HT22 cells, HT22 cells were seeded at 5 × 10^5^ cells/mL in 60 mm dishes and incubated overnight. The next day, the cells were treated with 5 μM IAA for 2 h and replaced with a medium containing 3 μg/mL gintonin for 24 h. The cells were treated with Ki16425, an LPA 1/3 receptor antagonist, or BAPTA-AM, a Ca^2+^ chelator, 30 min before gintonin treatment and co-treated with 3 μg/mL gintonin for 24 h. The medium was collected to prepare a sample and centrifuged (14,000 rpm, 5 min) to remove cell debris. The concentration of BDNF released was measured using a human BDNF pre-coated ELISA kit (PeproTech, Rocky Hill, NJ, USA), according to the company’s instructions. Absorbance was recorded at 450 nm using a microplate reader (Molecular Devices, San Jose, CA, USA).

### 4.6. Measurement of Phosphorylation of TrkB Receptors in HT22 Cells

To measure the phosphorylation of TrkB receptors using an experimental assay, the HT22 cells were seeded at 5 × 10^5^ cells/mL in 60-mm dishes and incubated overnight. Before this assay, the cell culture medium was replaced with serum-free DMEM for 4 h, and the cells were treated with the replaced medium in the presence or absence of 3 μg/mL gintonin or 30 ng/mL BDNF for 24 h at 37 °C. The level of TrkB receptor phosphorylation in cells was measured using the PathScan^®^ Phospho-TrkB ELISA Kit (Cell Signaling Technology, Beverly, MA, USA), according to the manufacturer’s instructions. All samples were subjected to Western blot analysis after protein quantification.

### 4.7. Western Blot Analysis

After drug treatment of HT22 cells grown in 60-mm dishes, the cells were washed with ice-cold PBS and lysed with 100 μL of radioimmunoprecipitation assay (RIPA) lysis buffer (150 mM NaCl, 0.25% sodium deoxycholate, 1 mM EGTA, 1% NP-40, and 50 mM Tris-HCl, pH 8.0). Lysates were incubated for 1 h on ice and then centrifuged at 14,000 rpm for 20 min. The supernatants were collected, and the protein concentration was determined using a BCA Protein Assay Kit (Thermo Fisher Scientific Korea, Gangnam-gu, Seoul, Korea). Twenty-five micrograms of protein were electrophoresed on a 10–12% SDS-PAGE gel and then transferred electrophoretically onto a 0.45 μm hydrophobic PVDF membrane (Millipore, MA, USA). The membranes were quenched with 5% BSA in TTBS for 1 h at room temperature on a shaker and incubated overnight at 4 °C with the following primary antibodies: primary anti-BDNF antibody (1:1000; Abcam, Cambridge, UK) and phospho-Akt (1:1000; Cell Signaling Technology, Danvers, MA, USA). The next day, after washing four times with TTBS, the membrane was incubated with a secondary antibody (HRP-conjugated anti-lgG antibody, anti-rabbit, GeneTax, Irvine, CA, USA) at a dilution of 1:1000 at room temperature for 2 h and exposed using Clarity Western ECL Substrate (Bio-Rad, Hercules, CA, USA).

### 4.8. Statistical Analysis

All experiments were repeated at least three times. Data are expressed as the mean ± standard error of mean (SEM). The mean values except BDNF level experiments were normalized as % of control. Differences among the groups were analyzed using one- or three-way analysis of variance, followed by a Dunnett’s test, and statistical significance was set at *p* < 0.05.

## Supplementary Materials

Figure S1: Effect of gintonin on ATP content in IAA-treated HT22 cells, Figure S2: Effect of gintonin on IAA-induced reduction in BDNF amount in HT22 cells, Figure S3: Effects of gintonin on [Ca^2+^]_i_ transients in HT22 cells.

## Sample Availability

Samples of the compounds are available from the authors.
