# Supplementary material for: Protective Effects of Gintonin on Reactive Oxygen Species-Induced HT22 Cell Damages: Involvement of LPA1 Receptor-BDNF-AKT Signaling Pathway
Source: Molecules. 2021 Jul 7;26(14):4138. doi: 10.3390/molecules26144138 (PMC8303475; doi:10.3390/molecules26144138)
Supplement: Supplementary file 1 [file molecules-26-04138-s001.zip › molecules-1275143-supplementary.pdf]

molecules

Supplementary Materials

Protective Effects of Gintonin on Reactive Oxygen Species-Induced HT22 Cell Damages: Involvement of LPA1 Receptor-BDNF-AKT Signaling Pathway

Yeon-Jin Cho [1], Sun-Hye Choi [1], Ra-Mi Lee [1], Han-Sung Cho [1], Hyewhon Rhim [2], Hyoung-Chun Kim [3], Byung-Joo Kim [4], Jong-Hoon Kim [5] and Seung-Yeol Nah [1,*]

[1] Ginsentology Research Laboratory and Department of Physiology, College of Veterinary Medicine, Konkuk University, Seoul 05029, Korea; yeonjin0202@naver.com (Y.-J.C.); vettman@naver.com (S.-H.C.); rmlee12@konkuk.ac.kr (R.-M.L.); newlove0202@nate.com (H.-S.C.)
[2] Center for Neuroscience, Korea Institute of Science and Technology, Seoul 02792, Korea; e-hrhim@kist.re.kr
[3] Neuropsychopharmacology and Toxicology program, College of Pharmacy, Kangwon National University, Chunchon 24341, Korea; kimhc@kangwon.ac.kr
[4] Division of Longevity and Biofunctional Medicine, Pusan National University School of Korean Medicine, Yangsan 50612, Korea; vision@pusan.ac.kr
[5] College of Veterinary Medicine, Biosafety Research Institute, Chonbuk National University, Baekje-daero 567, Jeonju-city 28644, Korea; jhkim1@jbnu.ac.kr
* Correspondence: synah@konkuk.ac.kr; Tel.: +82-2-450-4154

Supplementary Methods

Measurement of Intracellular ATP Levels

Intracellular ATP levels were determined using an ATP Assay Kit (BIOMAX, Nowon-Ku, Seoul, Korea) according to the manufacturer's instructions. Briefly, HT22 cells were seeded at 5 × 10^5 cells/mL in 60-mm dishes and incubated overnight. The next day, the cells were washed with serum-free DMEM and treated as indicated for the cell viability assay. For cell lysis, the cells in each dish were washed with cold phosphate-buffered saline (PBS), scraped, and resuspended in 100 μL of ATP assay buffer. Subsequently, the cells were centrifuged (14,000 rpm, 5 min), and the supernatant was transferred to a new tube and deproteinized using a Carrez Reagent Kit (BIOMAX, Nowon-Ku, Seoul, Korea). The reaction was initiated by adding 50 μL of the reaction buffer containing enzyme mix, converter, and probe to each microplate well containing 50 μL of standard or deproteinized samples. After incubation for 30 min at room temperature in the dark, fluorescence was measured using a Gemini-EM fluorescence microplate reader (Molecular Devices, San Jose, CA, USA) (Ex/Em = 535/587 nm).

Immunofluorescence Staining

For immunochemistry, HT22 cells were seeded at 2 × 10^5 cells/mL on 10 mm glass coverslips coated with PLL. The next day, the cells were treated with 5 μM IAA for 2 h. Then, the medium containing IAA was removed and replaced with medium without IAA but containing 3 μg/mL gintonin. After 24 h, the cells were fixed in 4% paraformaldehyde in PBS for 20 min at room temperature in the dark. Fixed cells were washed three times with PBS and permeabilized using 0.3% Triton X-100 in 3% bovine serum albumin (BSA) for 30 min at room temperature. The cells were incubated overnight at 4 °C with the following primary antibodies: primary anti-BDNF antibody (1:1000; Abcam, Cambridge, UK), phospho-Akt antibody (1:200; Cell Signaling Technology, MA, USA), and phospho-TrkB (1:200; Cell Signaling Technology, MA, USA). The next day, the cells were washed three times with PBS and incubated with goat anti-rabbit IgG (Alexa Fluor 488; Abcam, Cambridge, UK) diluted 1:1000 in BSA for 2 h at 37 °C in the dark. Following three washes with PBS, the cells were stained for nuclei and mounted with Vectashield Mounting Media (Vector Laboratories, Burlingame, CA,

USA) containing DAPI. Immunofluorescence images were obtained using an Axio200 inverted fluorescence microscope (Carl Zeiss, Oberkochen, Baden-Württemberg, Germany).

Measurement of $[Ca^{2+}]_i$ Transient Levels

To measure the intracellular calcium concentration, Fura 2-AM (Sigma-Aldrich, St. Louis, MO, USA), a calcium indicator, was used. HT22 cells were seeded in 10 cm dishes until they were 80-90% confluent. Then, the cells were treated with 5 μM IAA for 2 h or left untreated, separated by treatment with trypsin/EDTA, and washed with calcium HEPES-buffered solution containing 120 mM NaCl, 25 mM HEPES, 10 mM glucose, 5 mM KCl, 1.5 mM $CaCl_2$, and 1 mM $MgCl_2$, with the pH adjusted to 7.4 with NaOH. After washing, 10 μM Fura 2-AM was added to the cells, and the cells were incubated for 1 h at 37 °C in a shaker. To measure the intracellular calcium concentration of cell suspensions loaded with Fura 2-AM, an RF-5300PC intracellular ion measurement system (Shimadzu Corporation, Kyoto, Japan) was used (Ex/Em = 340 and 380/510 nm), as previously described [1,2].

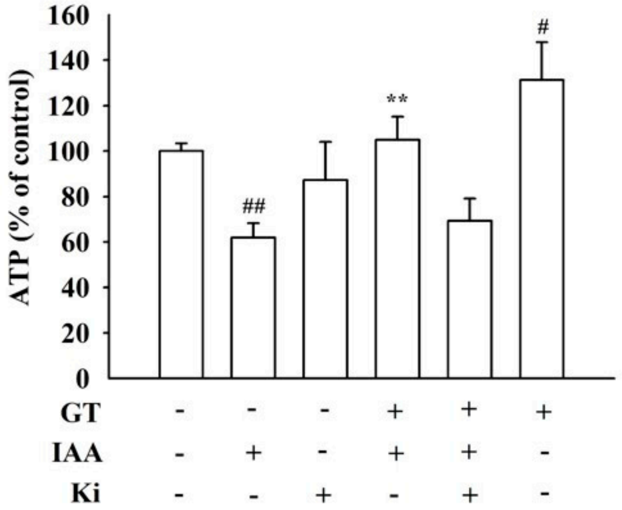

Figure S1. Effect of gintonin on ATP content in IAA-treated HT22 cells. HT22 cells were exposed to IAA (5 μM) for 2 h to induce ATP depletion. The cells were incubated with serum-free DMEM with or without gintonin (3 μg/mL) for 24 h. Gintonin increased ATP production after IAA treatment. Treatment with 10 μM Ki16425, an LPA1/3 receptor antagonist, for 30 min inhibited gintonin-mediated ATP production in HT22 cells. ATP levels were measured using an ATP kit. Data are expressed as the mean ± standard error of the mean (SEM; n = 4). # $p < 0.05$ and ## $p < 0.01$, compared to the control group; ** $p < 0.01$, compared to the IAA-treated group.

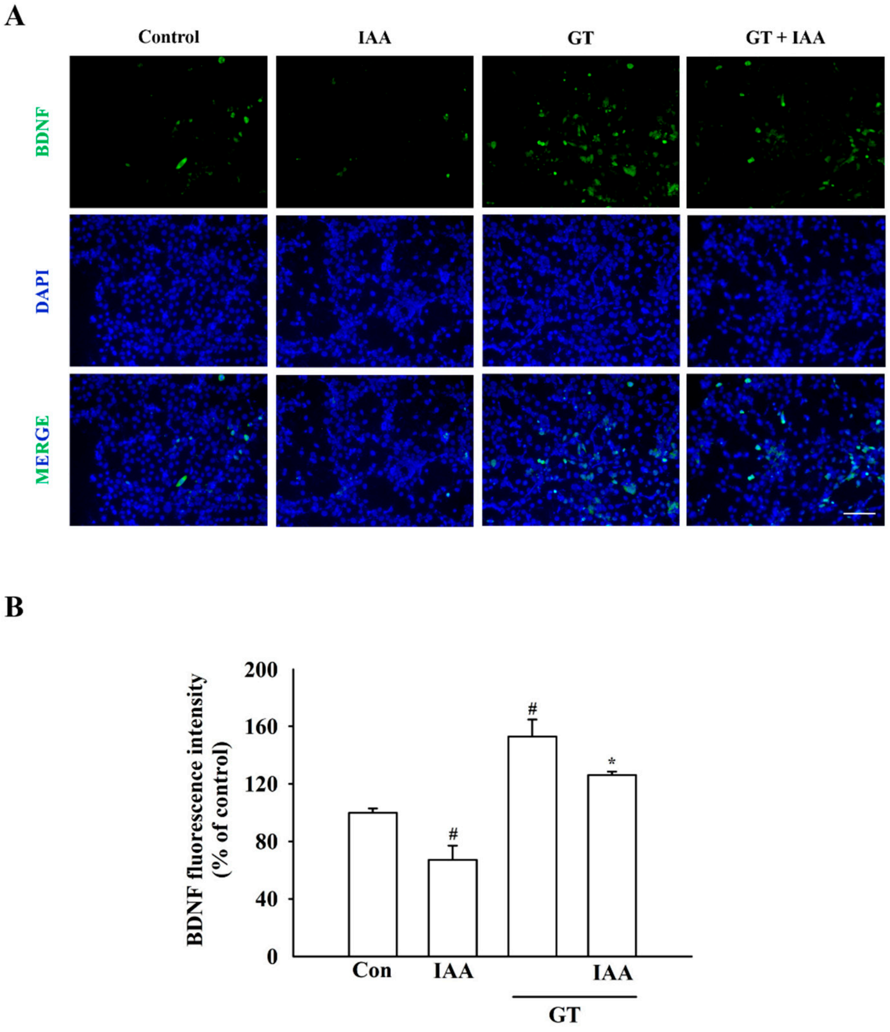

Figure S2. Effect of gintonin on IAA-induced reduction in BDNF expression in HT22 cells. (**A**) Representative microscopy images of BDNF expression. The expression of BDNF was detected using immunocytochemistry techniques. HT22 cells were treated with 5 μM IAA for 2 h, followed by gintonin (3 μg/mL) for 24 h. BDNF is dyed green, and nuclei are dyed blue using DAPI. Microscopy images were captured at the same magnification, scale bar = 100 μm. (**B**) Quantitative analysis of the images obtained from fluorescence microscopy. The data are expressed as the mean ± standard error of the mean (SEM; $n = 4$). $^{\#}$ $p < 0.05$ compared to the control group; ** $p < 0.01$ compared to the IAA-treated group.

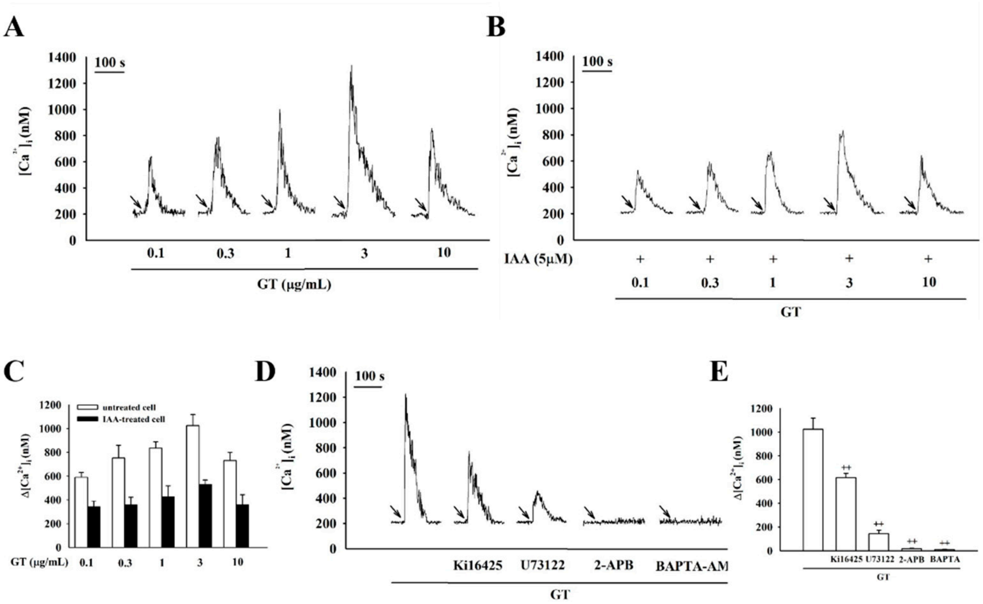

Figure S3. Effects of gintonin on $[Ca^{2+}]_i$ transients in HT22 cells. (**A**) Representative $[Ca^{2+}]_i$ transient traces obtained after treatment of HT22 cells with the indicated concentrations of gintonin. (**B**) Representative traces obtained after treatment of indicated concentrations of gintonin in the presence of IAA. (**A** and **B**) Gintonin treatment (0.1–10 μg/mL) induced $[Ca^{2+}]_i$ transients. (**C**) Histograms representing the dose responses of gintonin on $[Ca^{2+}]_i$ transients. (**D**) Representative traces of gintonin-mediated $[Ca^{2+}]_i$ transients in the absence or presence of various antagonists. The arrows represent the application of gintonin (3 μg/mL). The LPA1/3 receptor antagonist (Ki16425, 10 μM), phospholipase C (U73122, 5 μM), inositol 1,4,5-triphosphate receptor antagonist (2-APB, 100 μM), or the intracellular Ca^{2+} chelator (BAPTA-AM, 50 μM) was added to HT22 cells 30 min before gintonin treatment. (**E**) Histograms representing the effect of various antagonists on gintonin-mediated $[Ca^{2+}]_i$ transients. The data are expressed as the mean ± standard error of the mean (SEM; $n = 5$). $^{++}$ $p < 0.01$ compared to the gintonin-treated group.

References

1. Hwang, S.H.; Shin, T.J.; Choi, S.H.; Cho, H.J.; Lee, B.H.; Pyo, M.K.; Lee, J.H.; Kang, J.; Kim. H.J.; Park, C.W.; et al. Gintonin, newly identified compounds from ginseng, is novel lysophosphatidic acids-protein complexes and activates G protein-coupled lysophosphatidic acid receptors with high affinity. *Mol Cells*. **2012**, *33*, 151–162.
2. Pyo, M.K.; Choi, S.H.; Hwang, S.H.; Shin, T.J.; Lee, B.H.; Lee, S.M.; Lim, Y.H.; Kim, D.H.; Nah, S.Y. Novel Glycolipoproteins from Ginseng. *J Ginseng Res*. **2011**, 35, 92–103.
